# Evaluation of five diagnostic methods for *Strongyloides stercoralis* infection in Amhara National Regional State, northwest Ethiopia

**DOI:** 10.1186/s12879-022-07299-1

**Published:** 2022-03-28

**Authors:** Tadesse Hailu, Arancha Amor, Endalkachew Nibret, Abaineh Munshea, Melaku Anegagrie, Maria Delmans Flores-Chavez, Thuy-Huong Ta Tang, Jose M. Saugar, Agustín Benito

**Affiliations:** 1 Department of Medical Laboratory Science, College of Medicine and Health Sciences, Bahir Dar University, Bahir Dar, Ethiopia; 2 Mundo Sano Foundation and Institute of Health Carlos III, Madrid, Spain; 3 Biology Department, Science College, Bahir Dar University, Bahir Dar, Ethiopia; 4 Mundo Sano Foundation and National Centre for Microbiology, Institute of Health Carlos III, Madrid, Spain; 5 National Center of Tropical Medicine, Institute of Health Carlos III, Biomedical Research Networking Center of Infectious Diseases (CIBERINFEC), Madrid, Spain; 6 National Centre of Microbiology, Institute of Health Carlos III, Biomedical Research Networking Center of Infectious Diseases (CIBERINFEC), Madrid, Spain; 7 National Centre of Tropical Medicine, Institute of Health Carlos III, Biomedical Research Networking Center of Infectious Diseases (CIBERINFEC), Madrid, Spain

**Keywords:** Amhara Region, Diagnosis, Sensitivity, Specificity, *Strongyloides stercoralis*

## Abstract

**Background:**

*Strongyloides stercoralis* is an intestinal parasite that can cause chronic infection, hyperinfection and/or a dissemination syndrome in humans. The use of techniques targeting ova fails to detect *S. stercoralis*, as only larvae of the parasite are excreted in faeces. Due to the absence of “Gold” standard diagnostic method for *S. stercoralis*, there is a paucity of reported data worldwide.

**Objective:**

This study aimed to evaluate the performance of diagnostic methods of *S. stercoralis* infection by taking the composite reference as a “Gold” standard.

**Methods:**

A cross-sectional study was conducted among 844 schoolchildren in Amhara Region, Ethiopia, from April to December 2019. Stool samples were collected and processed with formol-ether concentration technique (FECT), spontaneous tube sedimentation technique (STST), Baermann concentration technique (BCT), agar plate culture (APC) and real-time polymerase chain reaction (RT-PCR). Sensitivity, specificity, positive predictive value, and negative predictive value of each diagnostic method were computed against the composite reference. The agreements of diagnostic methods were evaluated by Kappa value at 95% CI.

**Results:**

The composite detection rate of *S. stercoralis* by the five diagnostic methods was 39.0% (329/844). The detection rate of the parasite from stool samples by FECT, STST, BCT, APC and RT-PCR was 2.0% (17/844), 4.0% (34/844), 10.2% (86/844), 10.9% (92/844) and 28.8% (243/844), respectively. The highest detection rate (37.8%; 319/844) of *S. stercoralis* was recorded by a combination of BCT, APC, and RT-PCR followed by a combination of STST, BCT, APC and RT-PCR (37.3%; 315/844). The sensitivity of FECT, STST, BCT, APC and RT-PCR against the composite reference was 5.2%, 10.3%, 26.4%, 28.0% and 73.9%, respectively. The diagnostic agreements of RT-PCR, APC, BCT, STST and FECT with the composite reference in detection of *S. stercoralis* were substantial (0.775), fair (0.321), fair (0.305), slight (0.123), and slight (0.062), respectively.

**Conclusion:**

RT-PCR detected the highest number of *S. stercoralis* infections. A combination of RT-PCR with APC and/or BCT better detected *S. stercoralis* from stool samples compared to other combinations or single diagnostic methods. Therefore, RT-PCR and combination of RT-PCR with APC and/or BCT diagnostic methods should be advocated for detection of *S. stercoralis* infection.

## Introduction

*Strongyloides stercoralis* is an intestinal nematode that causes strongyloidiasis in the tropics and subtropics. Based on estimates using newer diagnostic techniques-new immunoassays and molecular tests, about 370 million people were infected with *S. stercoralis* worldwide [1]. Infection by *Strongyloides* is classified as: sporadic (< 1%), endemic (1–5%) and hyperendemic (> 5%) [2].

The most accurate diagnosis for *S. stercoralis* infection is the detection of larvae in the stool [2]. Those diagnostic methods which have good sensitivity for ova detection of the parasites have almost nil sensitivity for *S. stercoralis* larvae detection, especially during chronic infection [3]. Among the methods used for the detection of eggs from stool specimens, direct saline microscopy [4], FECT [5] and STST [6] have the lowest sensitivity. The use of these methods for *S. stercoralis* detection might lead to misdiagnosis and underreporting of strongyloidiasis compared with BCT [7], APC [8–11], and RT-PCR or a combination of APC and BCT which have better detection rates [12]. Because of the low sensitivity of each method for larvae detection, a combination of methods is the most sensitive approach.

The two most sensitive parasitological methods for the diagnosis of *S. stercoralis* infection are BCT and APC, but they are not currently used as routine diagnostic techniques in health facilities in endemic countries [13]. A combination of these two methods is more sensitive than RT-PCR alone [12]. As a result, underdiagnosing and underreporting of *S. stercoralis* infection in endemic countries like Ethiopia is a common phenomenon [14]. Moreover, various scholars have reported variation in the sensitivity of diagnostic methods used for the detection of *S. stercoralis.* Therefore, this study aimed to explore the best approach for *S. stercoralis* diagnosis, by checking the performance of FECT, STST, BCT, APC, and RT-PCR against the composite reference in Amhara Region, northwest Ethiopia.

## Materials and methods

### Design, area and period of the study

A cross-sectional study was conducted among schoolchildren in Amhara Region from April to December 2019 to evaluate the performance of FECT, STST, BCT, APC and RT-PCR against the composite reference for the detection of *S. stercoralis*. Primary schoolchildren aged from 6 to 14 years, volunteered to provide stool samples and whose parents gave consent for participating were included. Thirteen primary schools were randomly selected in seven districts of Amhara Region. The total number of students attending 13 schools during the study period was 9509. Then, eight hundred forty-four schoolchildren were randomly selected from the 13 schools and were screened for *S. stercoralis* infection. Those schoolchildren who had taken anthelmintic drugs 3 months prior to data collection time were excluded from the study.

### Laboratory procedures

About 23 g of fresh stool sample were collected in stool cups (one time-collection) from each study participant and transported to the nearby health institution laboratory. The stool samples, fresh and unrefrigerated, were processed by FECT, STST, BCT, APC, and RT-PCR to detect *S. stercoralis* infection. Those schoolchildren who were found to be positive for *S. stercoralis* by any one of the above five diagnostic tests were considered as positive.

For the FECT, about half a gram of stool was processed in a concentration device which was based on modified Ritchie’s method (Young et al.). The device, Bioparaprep®, consists of a collection tube, a filtration unit and a concentration conical tube. Two and half milliliters of 10% formalin and one milliliter of ethyl acetate were added. The sediment was transferred to a slide and observed with a microscope to detect *S. stercoralis* larvae [15].

For the STST, approximately three grams of stool sample were homogenized in 10 ml of saline solution. The mixture was filtered through surgical gauze into a falcon tube which was then filled with more saline solution, plugged and shaken vigorously and finally it was left to stand for 45 min. The sediment was transferred to a slide and observed with a microscope to detect *S. stercoralis* larvae [6].

For the BCT, approximately 10–15 g of stool were mixed with water and powdered charcoal and then transferred to a petri dish and incubated. The stool sample was suspended in a funnel containing warm water and sieve connected to a rubber tube. The filtrate coming to the rubber tube was collected and then the sediment was examined with a microscope, first using 4×, then with 10× and 40× objectives to detect *S. stercoralis* larvae [16, 17].

For the APC, about three grams of stool were placed on the center of a petri dish containing nutrient agar and then, it was sealed. The surface of the agar-plate was analyzed daily with dissection microscope or visually with naked eyes. When furrows/tracks of moving larvae were detected [9], 5 mL of 10% formalin solution were added to the surface of agar plate and the solution was transferred to a conical tube. The sediment was then observed with a microscope. Identification of *S. stercoralis* and other parasites was done by observing the key diagnostic features of buccal cavity of rhabditiform larvae and tail region of filariform larvae [7, 8].

For real-time PCR, 180 to 200 mg of concentrated stool sample were used and DNA was extracted with QIAamp® DNA stool mini-kit (Qiagen, Hilden, Germany), following the manufacturer’s instructions [17]. Amplification of 18S ribosomal ribonucleic acid small subunits of *S. stercoralis* was done using specific primers (Forward primer: 5′-GAA TTC CAA GTA AAC GTA AGT CAT TAG C-3′; Reverse primer: 5′-TGC CTC TGG ATA TTG CTC AGT TC-3′) [18]. The final volume of the reaction was 25 μL, comprising 12.5 μL QUANTIMIX EASY kit (Biotools®) (dNTPs, PCR buffer, and Taq DNA), 0.5 μL of each forward and reverse, primers, 0.15 μL SYBR green, 6.35 μL water, and 5 μL DNA. Two positive controls (*S. stercoralis and S. venezuelensis*), one negative control, and one blank (sterilized water) were used. An initial denaturation step was run at 95 °C for 15 min, followed by 50 cycles made of denaturation at 90 °C for 10 s, annealing at 60 °C for 10 s, extension at 72 °C for 30 s and a final extension at 70 °C for 10 min. Amplification and fluorescence detection of the target gene of *S. stercoralis* was performed on a Corbett Rotor-Gene™ 6000 RT-PCR cycler (QIAGEN®, Hilden, Germany). The specificity of amplified products was assessed by melting curve analysis, which was done with a Rotor Gene™ 6000 Series software version 1.7 [19].

### Performance evaluation of diagnostic methods

To evaluate the performance of FECT, STST BCT, APC and RT-PCR for detection of *S. stercoralis*, sensitivity (SN), specificity (SP), positive predictive value (PPV), and negative predictive value (NPV) were calculated against the composite reference. The diagnostic agreement between a pair of methods was evaluated by Kappa value, number of observed agreements, a number of agreements expected by chance, and standard error. Kappa result was interpreted as follows: values ≤ 0 as no agreement; 0.01–0.20 as none to slight; 0.21–0.40 as fair; 0.41–0.60 as moderate; 0.61–0.80 as substantial; and 0.81–1.00 as almost perfect agreement [20].

### Data quality assurance and analysis

Prior to stool sample collection, training in the stool sample collection, diagnosis and results interpretation were given to laboratory personnel. Proper labeling of the stool cup with serial numbers was done. The amount of stool sample was checked during stool sample collection and transported to the nearby health institution laboratories within 30 min without any preservation method. Each test was performed by following standard operating procedure. To eliminate observer bias, stool slides were examined independently by two laboratory technologists and the results of their observations were recorded on separate sheets for later comparison. The discordant results were re-checked by the principal investigator. Generally, the data quality assurance was checked during pre-analytical, analytical and post analytical stages of the laboratory process [8, 21].

Data were entered into EpiData software and analyzed by Statistical Package for Social Sciences (SPSS) version 23 statistical software. Prevalence of *S. stercoralis* infection was calculated by descriptive statistics. The SN, SP, PPV and NPV of each diagnostic method for *S. stercoralis* infection against the composite reference were calculated by frequency distribution. The diagnostic agreements of diagnostic methods were computed by Kappa value at 95% CI.

### Ethical approval and consent to participate

Ethical clearance was secured from the Ethical Review Committee of Science College, Bahir Dar University (Ref. Nº: PGRCSVD/149/2011). Permission letters were also obtained from the Amhara National Regional Health Bureau, Amhara National Regional Education Bureau, Zonal and District Education Offices. Informed consent was obtained from the parents by health extension workers, after explaining the purpose and objective of the study. The study was carried out in accordance with relevant guidelines and regulations (Declaration of Helsinki). The study participants’ laboratory results were kept confidential. Permits for exporting DNA for molecular analyses in Spain were obtained from the Ethiopian Biodiversity Institute in Addis Ababa (Ref. Nº: EBI71/1769/2020). Study participants who were positive for soil-transmitted helminths (STHs) and *S. stercoralis* were treated with albendazole and ivermectin, respectively. Other study participants infected with other types of intestinal parasites were linked to medical staff of the nearby health institution for treatment.

## Results

### Socio-demographic characteristics and parasitic infections of study participants

In this study, 844 schoolchildren with a mean age of 10.3 years (age range: 6–14 years) and a standard deviation of 1.77 were included. Most of the study participants, 43.1%, were in the age group of 10–11 years, followed by 30.1% in 6–9 years age group. Male students accounted for 51.7% and most, 88.3%, were rural dwellers.

High prevalence of *S. stercoralis* was found among 12–14 age group, 48.2% (109/226), male participants, 45.0% (196/436), and rural dwellers 39.6% (295/745). Parasites identified other than *S. stercoralis* were hookworm species 33.2% (277/844), *Entamoeba histolytica/dispar* 23.8% (201/844), *Schistosoma mansoni* 20.4% (172/844), *Giardia duodenalis* 7.4% (62/844), *Ascaris lumbricoides* 4.5% (38/844), *Hymenolepis nana* 4.1% (35/844), *Enterobius vermicularis* 0.8% (7/844), *Trichuris trichiura* 0.7% (6/844), *Taenia* spp. 0.5% (4/844), and *Fasciola* spp. 0.4% (3/844)*.*

### Detection rates of *Strongyloides stercoralis* by different diagnostic methods

The prevalence of *S. stercoralis* using the five diagnostic methods was 39.0% (329/844) (Table 1). When the analysis was used single diagnostic method, the highest prevalence rate was found by RT-PCR, 28.8% (243/844), followed by APC, 10.9% (92/844), and BCT, 10.3% (87/844). The detection rate of *S. stercoralis* by RT-PCR was 7.1 and 14.3 times higher when compared with STST and FECT. These two methods also had a low detection rate compared with APC and BCT (Table 1). When a combination of BCT, APC and RT-PCR was employed, the prevalence of *S. stercoralis* was found to be 37.8% (319/844). Likewise, when a combination of STST, APC and RT-PCR was used, a prevalence of 37.2% (314/844) was recorded. When other approaches using four, or even two methods such as PCR and another more sensitive parasitological technique were combined and used for diagnosis, almost similar results were obtained (Table 1).

**Table 1:** Detection rate of *S. stercoralis* by FECT, STST, BCT, APC and RT-PCR and their combinations in stools of 844 school children from Amhara Region, northwest Ethiopia

No	Diagnostic methods	*S. stercoralis*	
		Pos N, (%)	Prevalence (95% CI)
1	FECT	17 (2.0)	1.26–3.20
2	STST	34 (4.0)	2.90–5.58
3	BCT	87 (10.3)	8.44–12.54
4	APC	92 (10.9)	8.97–13.18
5	RT-PCR	243 (28.8)	25.84–31.94
6	FECT ∪ STST	42 (5.0)	3.71–6.66
7	FECT ∪ BCT	93 (11.0)	9.08–13.31
8	FECT ∪ APC	98 (11.6)	9.62–13.95
9	FECT ∪ RT-PCR	252 (29.9)	26.87–33.03
10	STST ∪ BCT	98 (11.6)	9.62–13.95
11	STST ∪ APC	107 (12.7)	10.6–15.10
12	STST ∪ RT-PCR	272 (32.2)	29.16–35.46
13	BCT ∪ APC	115 (13.6)	11.48–16.11
14	BCT ∪ RT-PCR	296 (35.1)	31.92–38.35
15	APC ∪ RT-PCR	304 (36.0)	32.85–39.32
16	FECT ∪ STST ∪ BCT	103 (12.2)	10.16–14.58
17	FECT ∪ STST ∪ APC	111 (13.2)	11.04–15.60
18	FECT ∪ BCT ∪ APC	118 (14.0)	11.80–16.48
19	FECT ∪ STST ∪ RT-PCR	264 (31.3)	28.24–34.49
20	FECT ∪ BCT ∪ RT-PCR	299 (35.4)	32.28–38.72
21	FECT ∪ APC ∪ RT-PCR	309 (36.6)	33.43–39.91
22	STST ∪ BCT ∪ APC	124 (14.7)	12.46–17.24
23	STST ∪ BCT ∪ RT-PCR	304 (36.0)	32.85–39.32
24	STST ∪ APC ∪ RT-PCR	314 (37.2)	44.04–50.76
25	BCT ∪ APC ∪ RT-PCR	319 (37.8)	34.00–40.51
26	FECT ∪ STST ∪ BCT ∪ APC	127 (15.0)	12.8–17.62
27	FECT ∪ STST ∪ BCT ∪ RT-PCR	309 (36.6)	34.43–39.91
28	FECT ∪ STST ∪ APC ∪ RT-PCR	299 (35.4)	32.2–38.8
29	FECT ∪ BCT ∪ APC ∪ RT-PCR	321 (38.0)	34.82–41.36
30	STST ∪ BCT ∪ APC ∪ RT-PCR	315 (37.3)	34.12–40.63
31	FECT ∪ STST ∪ BCT ∪ APC ∪ RT-PCR	329 (39.0)	35.75–42.31

*Pos* positive, *FECT* formol-ether concentration test, *STST* spontaneous tube sedimentation technique, *BCT* Baermann concentration technique, *APC* agar plate culture, *RT-PCR* real time polymerase chain reaction, *U* union

### Performance of diagnostic methods for *Strongyloides stercoralis* detection

By using a combination of all the five diagnostic methods as composite reference, higher SN (73.9%), SP (100%), and NPV (16.8%) were obtained by RT-PCR followed by APC with SN (28.0%), SP (100%) and NPV (6.8%). FECT showed the lowest sensitivity (5.2%) and NPV (5.3%). The agreements of RT-PCR, APC, BCT, STST and FECT with the composite reference were substantial (0.775), fair (0.321), fair (0.305), slight (0.123), and slight (0.062), respectively (Table 2).

**Table 2:** Diagnostic performance of FECT, STST, BCT, APC and RT-PCR in *S. stercoralis* detection against the composite reference

Methods			SN (95% CI)	SP (95% CI)	PPV (95% CI)	NPV (95% CI)	Kappa value	χ^2^, *p*-value
	Composite reference							
	Pos (N)	Neg (N)						
FECT								
Pos	17	0	5.2 (3.0–8.14)	100 (99.3–100)	100.00	5.3 (5.1–5.4)	0.062	27.2, 0.000
Neg	312	515						
STST								
Pos	34	0	10.3 (7.3–14.1)	100 (99.3–100)	100.00	5.5 (5.4–5.7)	0.123	55.5, 0.000
Neg	295	515						
BCT								
Pos	87	0	26.4 (21.8–31.6)	100 (99.3–99.5)	100.00	6.7 (6.3–7.1)	0.305	151.4, 0.000
Neg	242	515						
APC								
Pos	92	0	28.0 (23.2–33.2)	100 (99.3–100)	100.00	6.8 (6.4–7.3)	0.321	161.6, 0.000
Neg	237	515						
RT-PCR								
Pos	243	0	73.9 (68.8–78.5)	100 (99.3–100.0)	100.00	16.8 (14.4–19.5)	0.775	534.2, 0.000
Neg	86	515						

*Pos* positive, *Neg* negative

### Diagnostic agreement between two methods in the detection of *Strongyloides stercoralis*

The diagnostic agreement between RT-PCR and each APC, BCT, STST and FECT was slight with Kappa values of 0.032, 0.031, 0.033 and 0.025, respectively. The measure of inter-rater reliability of APC with FECT, STST and BCT was slight (0.193), fair (0.292) and substantial agreement (0.680), respectively. The agreement of BCT with FECT and that of STST was fair 0.251 and 0.34, respectively. The Kappa value between STST and FECT was fair (0.375). The number of observed agreements was the highest between STST and FECT (96.3%; 813/844), followed by APC and BCT (94.0%; 793/844) and BCT and STST (91.1%; 769/844) (Table 3).

**Table 3:** Diagnostic agreement between any of the two methods (FECT, STST, BCT, APC, RT-PCR) used for the detection of *S. stercoralis* from stool specimens

Methods			Kappa value (95% CI)	NOA N, (%)	NAEC N, (%)	SEK	χ^2^, p-value
	Pos	Neg					
	RT-PCR						
FECT							
Pos	8	9	0.025 (0.009–0.059)	600 (71.1)	593.8 (70.35	0.017	2.8, 0.083
Neg	235	592					
STST							
Pos	14	20	0.033 (0.011–0.076)	595 (50.5)	586.6 (69.5)	0.022	2.65, 0.078
Neg	229	581					
BCT							
Pos	34	209	0.031 (0.003–0.124)	582 (69.0)	564.1 (66.8)	0.031	0.50, 0.019
Neg	53	548					
APC							
Pos	31	61	0.032 (0.027–0.091)	571 (67.7)	562.0 (66.6)	0.030	1.2, 0.271
Neg	212	540					

	APC						
FECT							
Pos	12	5	0.193 (0.093–0.292)	759 (89.93)	738.7 (87.52)	0.051	63.6, 0.000
Neg	80	747					
STST							
Pos	21	13	0.292 (0.185–0.398)	760 (90.1)	725.4 (86.0)	0.054	94.4, 0.000
Neg	71	739					
BCT							
Pos	64	23	0.680 (0.599–0.763	793 (94.0)	684.0(81.0)	0.042	392.2, 0.000
Neg	28	729					

	BCT						
FECT							
Pos	13	4	0.251 (0.136–0.367	759 (91.8)	736.2(89.0)	0.059	82.1, 0.000
Neg	74	753					
STST							
Pos	23	11	0.342 (0.231–0.453)	769 (91.1)	730.0 (86.49)	0.057	126.0, 0.000
Neg	64	746					

	STST						
FECT							
Pos	10	7	0.375 (0.204–0.547)	813 (96.3)	794.4 (94.1)	0.088	134.7; 0.000
Neg	24	803					

*NOA* number of observed agreements, *NAEC* number of agreements expected by chance, *SEK* standard error of Kappa, *Pos* positive, *Neg* negative

## Discussion

The absence of “Gold” standard diagnostic method and the employment of diagnostic methods normally used for the detection of eggs of other helminth parasites for the diagnosis of *S. stercoralis* infection tends to under diagnose and/or under-report strongyloidiasis in endemic areas [3]. In the present study, the detection rate of *S. stercoralis* by FECT was low (2.0%), even though it is slightly higher than 0.7% previously reported from Ethiopia [22] and 0.9% from Ghana [23]. It is also comparable to former reports 3% from northwest Ethiopia [24] and 1.99% from Nigeria [25]; however, it is lower than previous reports 5.8% from southern Ethiopia [26], 3.5% from northwest Ethiopia [17] and 13.13% from Nigeria [27]. Similarly, the detection rate of *S. stercoralis* by STST in the present study was 4.0%, which is lower than 16% [28] and 7% [6] from Peru. The observed difference between studies might be justified by intermittent excretion of the larvae in stool, variation in sample size, endemicity of *S. stercoralis*, geographical location (highland to lowland in the current study), age of the study participants, variation in interpersonal detection skills and the amount of time spent for searching larvae in microscopic fields.

The detection rate of *S. stercoralis* was increased by 2.7-fold when it was diagnosed with BCT as compared with FECT [13]. In the current study, the detection rate of *S. stercoralis* by BCT was 10.31%, which is consistent with 12.1% previously reported from northwest Ethiopia [17]. The current finding is, however, higher than an earlier report, 7.1%, from Tanzania [29], but it is lower than 19% from Côte d’Ivoire [30] and 34.5% in rural community of Ethiopia [31]. Variation in the detection rate of the parasite might be associated with low larva load, sample size difference, the degree to which laboratory technologists/researchers adhere to BCT procedure, and the difference in the level of endemicity of *S. stercoralis* infection in different geographical locations.

The detection rate of *S. stercoralis* by APC is higher than other traditional methods [32]. In this study, the detection rate of *S. stercoralis* by APC was 10.9%, which is consistent with 13.1% in Japan [11], but it is lower than 17.14% in Bangkok, Thailand [33]. The possible justification might be due to a difference in the daily petri dish examination, especially, in case of low parasitic load, and the employment of both visual inspections (track of the larvae on APC) and microscopy that increased the chance of *S. stercoralis* detection, geographical location, and the time spent for searching larvae in microscopic fields.

A recent study showed that the detection rate of *S. stercoralis* could be increased when RT-PCR was used instead microscopy based methods [34, 35]. In the present study, the detection rate of *S. stercoralis* by RT-PCR was 28.8%, which is lower than 36.2% previously reported [28], but it is higher than 13.4% in northwest Ethiopia [17], 6% in northern Australia [36], 25% in Lao People’s Democratic Republic [12]. The variation might be related to accuracy in DNA extraction, preservation and its amplification technique. Also, it might be related to the intensity of infection at the time of DNA extraction.

A combination of traditional methods increases the detection rate of *S. stercoralis* [13, 17]. Similarly, in the present study, the detection rate was increased by combining two or more diagnostic methods. This finding is supported by a previous report in Ethiopia [37]. Small number of larvae and an intermittent excretion of *S. stercoralis* larvae in the stool lead in false negative result. There is no a single technique with high sensitivity. An increased detection of the parasite was observed due to the employment of combined methods. By combining different detection methods, it was possible to increase the detection rate of *S. stercoralis* that would otherwise be missed if a single diagnostic technique were employed.

The detection rate of *S. stercoralis* by a combination of BCT and APC in this study was 13.6%, which is lower than 26% in Laos [12], but it is higher than 10.9% previously reported from Ethiopia [37]. Our finding is also comparable with 14.2% [38] and 11.7% [39] previously reported. Again, the difference might be due to the variation in the source of the sample, geographical location, and endemicity of the parasite.

Between three combined methods used in the present study, the detection rate of *S. stercoralis* by BCT, APC and RT-PCR was 37.8%, which is higher than 33.5% detection rate reported using the same combination [12]. Our current findings corroborate the fact that an increase in the *S. stercoralis* detection will be obtained if more than single diagnostic method is employed.

In this study, when a composite reference is used as a “Gold” standard, RT- PCR (73.9%) had the highest sensitivity followed by APC (28.0%) and BCT (26.4%). This finding is comparable with a previous report [12]. The sensitivity of the combined methods of APC and BCT in the present study was 19.5%, which is lower than 77.1% previously reported [12]. The difference in sensitivity might be due to low larval load and irregular excretion of larva in stool. When each parasitological technique was used, the sensitivity (5.2%) of FECT was found to be lower than 17.1% [17], 12.9% [31], and 47% [4] previously reported. Similarly, low sensitivity (10.3%) of STST was observed in the present study which is consistent with a previous report [6].

The sensitivity of BCT was 26.4%, which is lower than 88.9% [40] and 60% [12]. The variation in the size of stool sample used, intermittent excretion and the larvae load in the stool and the composite reference that was used as a “Gold” standard could be the possible justifications.

The sensitivity of APC, 28.0% which was found in the present study, is better than other larvae detection methods that are used for the diagnosis of *S. stercoralis* infection [38]. Our finding, 28.0%, is compared with 25% previous sensitivity report [41]. However, our finding is lower than earlier sensitivity reports, 100% [40] and 60% [12].

The sensitivity of RT-PCR in this study was 73.9%, which is compared with 74.3% [12] and 75% previous sensitivity reports [41]. Our result is, however, higher than 64.6% [16] and 65.1% [31] previously reported by the same method, but it is lower than 93.8% [19]. The difference might be due to low larval load, low larval excretion in chronic cases, and the type of composite reference that was used as a “Gold” standard. Although RT-PCR has high sensitivity for *S. stercoralis* detection in clinical services, this diagnostic method is rarely available in resource poor areas, due to its high cost.

The agreement between FECT and the APC was 0.193 which is lower than (0.668) substantial agreement [37]. The test agreement between BCT and RT-PCR, and that of APC and RT-PCR was also 0.031 and 0.032, respectively, and they were considered as slight agreements, which disagree with the previous report [12]. However, the agreement between BCT and APC (0.680) and that of RT-PCR and composite reference (0.775) were substantial which is consistent with a previous report [12], but it’s higher than the previous fair (0.140) agreement [41]. The variation might be due to the employment of egg detection diagnostic methods and the type of composite reference used. The number of stool samples collected, fecal amount and/or fecal dilution might also affect their detection power and sensitivity of methods used for the diagnosis of *S. stercoralis* infection. The use of single stool sample from each study participant for comparison of detection rates and evaluation of the performance of each diagnostic method might be the limitation of the study.

## Conclusion

The sensitivity of RT-PCR is higher than single microscopic diagnostic method used. Combining RT-PCR with BCT and/or APC gives a higher detection rate of *S. stercoralis* compared with a single microscopic method. Among the microscopic detection methods, a combination of BCT and APC shows the highest detection rate. The agreements between RT-PCR and composite reference and that of between APC and BCT were substantial. Therefore, RT-PCR and a combination of RT-PCR with BCT and APC or with APC should be used. A combination of APC and BCT is also recommended where there is no RT-PCR in poor areas as a better diagnostic approach.

## Data Availability

All data generated or analyzed during this study are included in this published article.
